# Critical parameters of fungal pathogenicity at the epithelial interphase

**DOI:** 10.1093/femsml/uqag001

**Published:** 2026-02-12

**Authors:** Anna Möslinger, Stefanie Allert, Slavena Vylkova, Bernhard Hube

**Affiliations:** Department of Microbial Pathogenicity Mechanisms, Leibniz Institute for Natural Product Research and Infection Biology - Hans-Knöll-Institute, 07745 Jena, Germany; Department of Microbial Pathogenicity Mechanisms, Leibniz Institute for Natural Product Research and Infection Biology - Hans-Knöll-Institute, 07745 Jena, Germany; The Benioff Center for Microbiome Medicine, University of California San Francisco, CA 94143 San Francisco, CA, United States; Department of Microbial Pathogenicity Mechanisms, Leibniz Institute for Natural Product Research and Infection Biology - Hans-Knöll-Institute, 07745 Jena, Germany; Faculty of Biological Sciences, Institute of Microbiology, Friedrich Schiller University Jena, 07743 Jena, Germany; Cluster of Excellence Balance of the Microverse, Friedrich Schiller University Jena, 07743 Jena, Germany

**Keywords:** *Candida albicans*, *Aspergillus fumigatus*, epithelium, adhesion, invasion, damage

## Abstract

Modern medical advances have contributed to a growing population of immunocompromised individuals, increasing susceptibility to human fungal pathogens. The leading species for invasive fungal diseases are *Candida albicans* and *Aspergillus fumigatus*. While *C. albicans* is a common colonizer of mucosal sites that can cause endogenous infections, *A. fumigatus* is an environmental fungus that typically infects individuals through the inhalation of spores. As a first point of contact, the epithelial cell layers serve both as a physical barrier and a sentinel to alert the immune system. Numerous fungal and host-derived mediators have been described that facilitate fungal adherence, invasion into epithelial cells, and subsequent host tissue damage. In cases of systemic infection, this can lead to fungal dissemination. This review focusses on the early interactions—specifically adhesion and invasion—between epithelial cells and the fungal pathogens *C. albicans* and *A. fumigatus*.

## Introduction

Despite their significant impact, human fungal pathogens are often overlooked in discussions of global health, yet they contribute substantially to morbidity and mortality worldwide (Brown et al. 2012). Superficial fungal infections affecting the skin and mucosa are among the most prevalent infectious conditions, but the emergence of life-threatening mycoses has raised serious concerns for public health. This alarming trend is closely tied to advances in modern medicine, e.g. chemotherapy, bone marrow and organ transplantation, as well as underlying conditions such as tuberculosis, influenza and HIV, or critically ill patients in the intensive care unit (ICU) (Köhler et al. 2017). These immunocompromised patients are particularly vulnerable to opportunistic fungal infections caused by *Candida, Aspergillus*, and *Cryptococcus* species (Hopke et al. 2018, WHO 2022). Among these, *Candida albicans* and *Aspergillus fumigatus* are the leading causative agents of invasive fungal diseases, driving severe morbidity and mortality in at-risk populations (Alcazar-Fuoli and Mellado 2014, Denning 2024). As the burden of fungal infections continues to rise, understanding the mechanisms by which these pathogens interact with their hosts remain critical for improving diagnostic and therapeutic strategies.

*Candida albicans* is a common colonizer of mucosal sites, such as the oral cavity, the vagina, and gastrointestinal tract (Ghannoum et al. 2010, Drell et al. 2013, Nash et al. 2017, Delavy et al. 2023, Katsipoulaki et al. 2024, Schille et al. 2025, Valentine et al. 2025). It is the most frequently isolated species in cases of candidiasis, accounting for ~45%–50% of these infections (Rodrigues et al. 2014). Moreover, *C. albicans* ranks among the leading causes of hospital-acquired infections, partly due to its ability to colonize medical devices such as catheters and prosthetics (Hopke et al. 2018). The global burden of invasive candidiasis is substantial, with an annual incidence of ~1 565 000 cases worldwide and a mortality rate of nearly 35% (Denning 2024).

*Aspergillus fumigatus* is an ubiquitous environmental filamentous fungus that is found in diverse ecological niches worldwide. Human infections with *A. fumigatus* typically occur *via* inhalation of its airborne spores, known as conidia (Köhler et al. 2017, Anandani et al. 2025). Conidia are present in indoor and outdoor environments, with concentrations ranging from 1 and 100 conidia per cubic metre under normal conditions. However, in specific niches, such as compost heaps or decaying organic matter, conidia concentrations can reach as high as 10^8^ conidia per cubic metre (Latgé and Chamilos 2019). Patients with an underlying condition such as chronic obstructive pulmonary disorder (COPD), various cancers, or those admitted to ICUs are at an increased risk of developing invasive aspergillosis. The annual prevalence of invasive aspergillosis is ~2 116 000 globally, with a mortality rate ranging from 43% to 72% even with treatment (Denning 2024). Chronic pulmonary aspergillosis (CPA), another manifestation of *A. fumigatus* infections, complicates pre-existing pulmonary diseases such as tuberculosis, COPD, or sarcoidosis. The annual incidence rate of CPA following tuberculosis infection is around 1 837 000 cases, with a treated mortality rate of around 8% (Denning 2024). However, these figures exclude patients who develop CPA secondary to other diseases, with the total number of affected individuals exceeding 3 million people worldwide (Kosmidis and Denning 2015). Allergic bronchopulmonary aspergillosis (ABPA), a hypersensitivity reaction to *A. fumigatus*, is a common complication in patients with asthma or cystic fibrosis. The prevalence of ABPA is estimated at 4.3% in diagnosed asthma cases, 4.5% in clinical asthma cases, and 8.6% in wheezing adults (Denning 2024). In addition to ABPA, 20%–30% of severe asthma patients show sensitization to fungal allergens, most commonly *A. fumigatus*, exacerbating disease outcome (Agarwal et al. 2023). These underscore the widespread impact of *A. fumigatus* on human health and the necessity for improved diagnostic and therapeutic strategies to address its diverse clinical manifestations.

Epithelial surfaces represent the primary host niches that come into contact with fungal pathogens such as *C. albicans* or *A. fumigatus*. As the first line of defence, epithelial cells play a pivotal role in maintaining tissue integrity and initiating immune responses. Hence, the interactions between epithelial cells and these fungal pathogens are highly dynamic and complex, with both species possessing critical factors modulating the outcome of host-pathogen interactions. This review focusses on the early interaction—specifically adhesion and invasion—between epithelial cells and the fungal pathogens *C. albicans* and *A. fumigatus*, and attributes that lead to epithelial damage.

## Candida albicans

### Adherence of *C. albicans* to epithelial cells

As a first line of defence, epithelial cells play a critical role in combating fungal pathogens by producing mucus, secreting antimicrobial agents—including antimicrobial peptides such as LL37, β-defensins, and cathelicidins—and releasing cytokines (Wasylnka et al. 2005, Naglik and Moyes 2011, Wächtler et al. 2012, Gao et al. 2015, Allert et al. 2018, Cortez and Schultz-Cherry 2021, Rowley et al. 2021). However, fungal pathogens have evolved virulence attributes to counteract these defence mechanisms. For example, mucus can inhibit *C. albicans* filamentation and hinder its direct adherence to the epithelial surface. However, *C. albicans* can overcome this barrier by proteolytically degrading the mucus layer through the secretion of secreted aspartic proteases (Sap) 2 (Colina et al. 1996, de Repentigny et al. 2000, Kavanaugh et al. 2014, Valle and Nobile 2020, Takagi et al. 2022). The response of epithelial cells to fungal pathogens, particularly cytokine secretion, has recently been reviewed in Mills et al. (2024).

**Figure 1:**
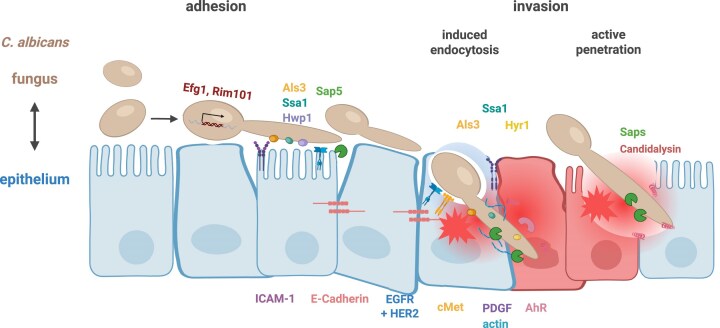
Epithelial and *C. albicans* mediators involved in adhesion and invasion. A mixture of different epithelial cell types is shown to represent different host niches. Fungal and host factors involved in adhesion, invasion, and host activities, as discussed in the text, are indicated. The symbols are shown in the same colours as their corresponding names and abbreviations.

Adherence to epithelial cell surfaces is the first step in direct fungal–host interaction, and multiple host and fungal factors have been identified as vital in this initial contact. On the host side, the epithelial glycoprotein intercellular adhesion molecule 1 (ICAM-1) is expressed in high glucose conditions and functions as a ligand for the adhesion of *C. albicans* (Egusa et al. 2005, Mikamo et al. 2018). Additionally, the transmembrane glycoprotein E-cadherin, which is highly expressed on epithelial cell surfaces, plays an important role in cell–cell adhesion by forming *trans*-homodimers within structures known as adherens junctions (Van den Bossche and Van Ginderachter 2013) (Fig. 1). Following stimulation with *C. albicans*, E-cadherin forms a multiprotein complex together with the epidermal growth factor receptor (EGFR) and the tyrosine kinase receptor c-Met (Phan et al. 2023). Several *C. albicans* gene products have been described to interact with members of this multiprotein complex, including proteins encoded by the *ALS* genes family, particularly Als3 (Phan et al. 2007, Wächtler et al. 2012, Phan et al. 2023), as well as Ssa1 (Sun et al. 2010, Phan et al. 2023, Qiu et al. 2023). In contrast, the fungal protease Sap5, regulated by Rim101, has been shown to degrade E-cadherin (Villar et al. 2007). Binding of *C. albicans* to this multiprotein complex induces cytoskeletal re-arrangements in epithelial cells and facilitates endocytosis of *C. albicans*, which will be further described below.

The fungal cell wall composition plays a critical mediator for the recognition of fungal cells by epithelial cells or immune cells patrolling epithelial tissues. The inner cell wall of most pathogenic fungi usually consists of chitin and β-1,3-glucan (Gow and Hube 2012, Gow et al. 2017, Hopke et al. 2018). Fungal β-1,3-glucan is recognized by the human C-type lectin receptor Dectin-1 (Brown et al. 2003), the lysin motif LYSMD3 (He et al. 2021) and ephrin type-A receptor 2 (EphA2) (Swidergall et al. 2017), while chitin is recognized by the toll-like receptor TLR9, the mannose receptor NOD2 and LYSMD3 (Wagener et al. 2014, He et al. 2021). To evade immune recognition, the outer cell wall of many pathogenic fungi masks these pathogen-associated molecular patterns (PAMPs) from host cells. This outer cell wall layer varies between fungal species and morphological stages. In *C. albicans*, it consists of mannosylated proteins (termed 'mannan') that cover the inner cell wall; notably, these structures are shorter and less dense in hyphae (Gow and Hube 2012, Lenardon et al. 2020). Genes regulating fungal cell wall composition are often directly involved in adhesion to epithelial cells. For example, the expression of the cell wall protein Eap1 enhanced the adherence of a yeast-locked mutant cells (*efg1*Δ/Δ) to kidney epithelial cells (Li and Palecek 2003). Another critical cell wall protein is the hypha-specific protein Hwp1, which is required for efficient adhesion to human buccal epithelial cells (Staab et al. 1999). Hwp1 binds to an epithelial cell surface protein mediated by a host transglutaminase. Interestingly, while Hwp1 is essential for *C. albicans* adhesion to oral epithelial cells, it is dispensable in disseminated candidiasis, highlighting the niche-specific relevance for host-fungus interaction (Staab et al. 2013). Therefore, fungal cell wall proteins are critical in facilitating the adherence of *C. albicans* to epithelial cells.

### *C. albicans* invasion into epithelial cells

Macrophages and neutrophils recognize PAMPs on fungal cells, triggering phagocytosis and phagosome maturation. This process is accompanied by the production of immune modulators and antimicrobial agents, such as reactive oxygen and nitrogen species, which typically lead to fungal clearance. However, in certain contexts, such as vulvovaginal candidiasis (VVC), this immune response can result in immunopathology (Fang 2004, Jimenez-Lopez et al. 2013, Austermeier et al. 2020, He et al. 2022, Zhao et al. 2022, Katsipoulaki et al. 2024, Schille et al. 2025, Valentine et al. 2025). Conversely, *C. albicans* invasion of epithelial cells occurs *via* two separate routes: epithelial endocytosis induced by hyphal-associated invasins or active penetration by hyphae and physical forces. Both invasion mechanisms result in the invagination of *C. albicans* and formation of an 'invasion pocket', where the epithelial membrane tightly surrounds the hyphae (Zakikhany et al. 2007, Dalle et al. 2010, Wächtler et al. 2012, Goyer et al. 2016, Mogavero et al. 2021), a process mediated in part by epithelial repair mechanisms (see below) (Westman et al. 2022). While these invasion processes resemble the phagocytosis performed by 'professional phagocytes', such as macrophages and neutrophils, they rely on distinct mechanisms and occur with different frequencies. For example, macrophages were shown to phagocytose 40% of *C. albicans* yeast cells (*via* recognition of cell wall components) at 1 h post infection (hpi) (Kasper et al. 2018). By comparison, oral epithelial cells, considered as 'nonprofessional phagocytes', internalize ~30% of germinating yeasts through invasins and induced endocytosis at 2 hpi (Dalle et al. 2010). Notably, the invasion capacity decreases as epithelial cells undergo differentiation, which correlates with the stage of formation of microvilli-like structures (Dalle et al. 2010, Goyer et al. 2016). For example, the *C. albicans* invasion rate decreases from ~30% in undifferentiated oral epithelial cells (three days post seeding) to about 10% in differentiated cells (23 days post seeding) (Dalle et al. 2010). Induced endocytosis is associated with an accumulation of actin microfilaments around the invading hyphae (Park et al. 2005), a phenomenon observed in *C. albicans* invasion of oral epithelial cells but not intestinal enterocytes (Dalle et al. 2010). Inhibition of induced endocytosis using actin inhibitors such as cytochalasin D, or infecting with killed hyphae (excluding active penetration), suggests that invasion of intestinal M cells, oral and vaginal epithelial cells likely occurs through a combination of active penetration and induced endocytosis (Fig. 1). In contrast, invasion of intestinal enterocytes exclusively relies on active penetration (Dalle et al. 2010, Wächtler et al. 2012, Goyer et al. 2016, Allert et al. 2018). Interestingly, disruption of tight junctions in differentiated intestinal enterocytes enables *C. albicans* endocytosis, albeit at a reduced rate (~8% at 2 hpi) (Goyer et al. 2016). These observations highlight the interplay between fungal invasion mechanisms and epithelial differentiation, as well as the importance of epithelial integrity in resisting fungal invasion.

### *C. albicans* internalization *via* induced endocytosis

Numerous *C. albicans* genes are implicated in both adherence to epithelial cells and induced endocytosis. For example, Als3–and to a lesser degree Als1 and Als5–bind to E-cadherin on epithelial cells, thereby stimulating actin-mediated endocytosis (Phan et al. 2007). In addition to E-cadherin, Als3 also interacts with EGFR, which forms a heterodimer with human epidermal growth factor receptor HER2 (also bound by Ssa1) and c-Met (also bound by Hyr1) (Zhu et al. 2012, Phan et al. 2023). While Hyr1 and Ssa1 function exclusively as invasins in induced endocytosis, Als3 might also contribute to active penetration (Wächtler et al. 2012, Phan et al. 2023). During *C. albicans* infection, E-cadherin operates within the same signalling pathway as EGFR and c-Met, together forming a multi-protein complex that mediates induced endocytosis of *C. albicans* by epithelial cells (Phan et al. 2023). Inhibition of either EGFR or c-Met alone results in a comparable reduction in *C. albicans* endocytosis; however, simultaneous inhibition of both results in a synergistic effect. While activation of EGFR with a natural ligand had no effect on endocytosis (Zhu et al. 2012), activation of c-Met with a natural ligand increased endocytosis (Phan et al. 2023).

In addition to this multiprotein complex, inhibition of the pattern recognition receptor (PRR) EphA2 reduces *C. albicans* endocytosis to a degree comparable to EGFR inhibition. EphA2 phosphorylation is stimulated by β-glucan present in the fungal cell wall, initiating signalling through the transcription factor STAT3. This signalling cascade results in the secretion of pro-inflammatory cytokines and host defence peptides. Notably, sustained EphA2 signalling requires both EGFR and factor(s) expressed by live *C. albicans* (Swidergall et al. 2017).

The cytokine IFN-γ has been shown to reduce *C. albicans* virulence and is currently being evaluated as a potential therapeutic option (Delsing et al. 2014, Gozalbo et al. 2014). In addition to reducing cytotoxicity, IFN-γ-treated epithelial cells have a reduced ability to endocytose *C. albicans*, although active penetration remains unaffected (Solis et al. 2017). Transcriptional profiling reveals that IFN-γ does not affect mRNA levels of key endocytosis receptors, such as EGFR, ERB2 (HER2), or CDH1 (E-cadherin). However, it markedly upregulates the rate-limiting enzyme in tryptophan catabolism *via* the kynurenine pathway, indoleamine 2,3-dioxygenase (IDO). In response to IFN-γ, IDO activity is stimulated, leading to tryptophan depletion and the production of kynurenines and intermediate metabolites. Kynurenines act as endogenous ligands for the cytoplasmic aryl hydrocarbon receptor AhR, which is essential for Src family kinases (SFK) activity. Prolonged SFK phosphorylation leads to reduced EGFR phosphorylation. Consequently, IFN-γ reduces endocytosis by promoting kynurenine production, thereby activating AhR and SFK, which ultimately downregulate the endocytosis receptor EGFR (Solis et al. 2017).

Host neural precursor-cell-expressed developmentally down-regulated protein 9 (NEDD9) and platelet-derived growth factor (PDGF) BB are upregulated during the early *C. albicans*-epithelial cell interaction (up to 8 h). Inhibition or knockdown of PDGF BB or NEDD9 reduces *C. albicans* endocytosis by epithelial cells in an E-cadherin-independent manner. Transcriptional profiling has revealed distinct infection site-specific activation for each protein: PDGF BB is activated during VVC in women, whereas NEDD9 is exclusively activated in disseminated infections (Liu et al. 2015).

### Invasion of *C. albicans* by active penetration of epithelial cells

During induced endocytosis, engulfed hyphae are surrounded by epithelial membrane protrusions, such as ruffles or filopodia (Zakikhany et al. 2007). However, these structures are absent when endocytosis is inhibited with cytochalasin D. Instead, invading hyphae exhibit invaginations and larger gaps between the penetrating hypha and the host membrane (Wächtler et al. 2012). In contrast, when epithelial cells are killed with 4% paraformaldehyde, invading hyphae are surrounded by disrupted cellular structures with no evidence of an intact host membrane (Wächtler et al. 2012). This shows that *C. albicans* can invade epithelial cells independent of epithelial cell factors or activities, similar to its invasion into inert media such as agar. Hyphae formation is critical for fungal invasion, as hypha-deficient mutants lacking the transcription factor Efg1 fail to invade host cells (Dieterich et al. 2002, Felk et al. 2002). Physical contact between *C. albicans* and epithelial cells strongly influences hyphae formation (Zakikhany et al. 2007). For example, a mutant lacking Eed1–a downstream target of Efg1 and key regulator of Ume6 (Martin et al. 2011)—produces short germ tubes but fails to sustain hyphal growth and form extended filaments in liquid media, instead growing as pseudohyphae on oral epithelial cells (Zakikhany et al. 2007, Martin et al. 2011). Similarly, *eed1*Δ/Δ mutant cells cannot penetrate agar but are taken up by epithelial cells *via* induced endocytosis, driven by transient expression of invasins (Zakikhany et al. 2007). While *eed1*Δ/Δ proliferate as yeast or pseudohyphae inside epithelial cells, they remain trapped intracellularly, unlike the parental strain. Thus, Eed1 is required not only for active penetration into epithelial cells but also for escape and intraepithelial dissemination (Zakikhany et al. 2007). Therefore, active tissue penetration requires both hyphal formation (Dieterich et al. 2002, Felk et al. 2002, Dalle et al. 2010) and hyphal extension (Zakikhany et al. 2007).

In addition to hyphae formation, secreted hydrolases—particularly secreted aspartic proteases (Sap)—are proposed mediators of *C. albicans* invasion and virulence (Naglik et al. 2003, Dalle et al. 2010, Katsipoulaki et al. 2024, Schille et al. 2025). The presence of *C. albicans* hyphae increases the expression of protease-activated receptors (PAR) on epithelial surfaces (Kumar et al. 2022). During infection, Saps proteolytically degrade extracellular mucus protein (Morschhäuser et al. 1997), as well as junctional proteins such as E-cadherin and zonula occludens 1 (ZO-1) (Colina et al. 1996, Villar et al. 2007, Kumar et al. 2022). There are multiple Sap antigens that are either found on both yeast and hyphae (Sap1-3) morphology or only on hyphae (Sap4-6) (Felk et al. 2002). While Sap1-3 do not appear to be involved in epithelial cell invasion, mutants lacking Sap4-6 (single, double or triple deletions) show a strong invasion deficit or even fail to invade host cells (Felk et al. 2002, Dalle et al. 2010). Similarly, treatment with the aspartic protease inhibitor pepstatin A reduced *C. albicans* invasion in a concentration-dependent manner (Dalle et al. 2010). This findings highlight the critical role of Sap proteases in *C. albicans* invasion, and their involvement in VVC has been recently reviewed by Bras et al. (2024).

### Deep invasion, repair, and damage

At later stages after initial invasion, *C. albicans* hyphae are enveloped by epithelial cell membranes, forming trans-cellular tunnels (TCTs) that allow the hyphae to cross multiple epithelial cells, with each membrane enveloping the invading hyphae. In intestinal epithelial cells, TCT formation during early invasion stages does not appear to be associated with epithelial damage; however, epithelial cells are damaged at later infection stages (Lachat et al. 2022). On the contrary, TCTs are not consistently formed during invasion of HeLa cells, and early interactions with *C. albicans* can be either damaging or nondamaging. Although the exact mechanisms underlying TCT formation remain to be elucidated (Lachat et al. 2022), epithelial repair processes are likely involved (Lapaquette et al. 2022, Westman et al. 2022).

For maximum host cell damage, the *C. albicans* peptide toxin candidalysin (Moyes et al. 2016) must be delivered into the invasion pocket (Mogavero et al. 2021). Candidalysin is secreted by hyphae and is embedded in the precursor protein Ece1. In addition to a candidalysin precursor, Ece1 also encodes seven noncandidalysin Ece1 peptides (NCEPs), which are required for proper intracellular folding (Müller et al. 2024). Correct folding of Ece1 is essential for inducing candidalysin pore formation and subsequent cellular epithelial cell damage (Russell et al. 2022, Müller et al. 2024). Despite its role in epithelial damage, candidalysin is not involved in epithelial cell invasion (Mogavero et al. 2021). Instead, candidalysin impairs barrier integrity, induces actin remodelling, and increases cell permeability, leading to the release of cytoplasmatic content, cytokines, and the antimicrobial peptide LL37 (Morelli and Queiroz 2025). Candidalysin appears to interfere with host glycosaminoglycan, and binding to glycosaminoglycan facilitates its enrichment on the host cell surface. Interestingly, the addition of exogenous sulphated glycosaminoglycans protects cells against candidalysin-induced damage (Lin et al. 2024). Additionally, candidalysin triggers an intracellular calcium influx, causing PtdIns(4,5)P_2_ hydrolysis and loss of cortical actin (Westman et al. 2022). To counteract the physical forces exerted by invading *C. albicans* hyphae and secreted candidalysin, epithelial cells utilize two repair mechanisms (Westman et al. 2022). Candidalysin is clustered into small vesicles formed by ALG-2 and ESCRT-III, whereas long invading hyphae trigger lysosome exocytosis, releasing lysosomal contents to repair the plasma membrane (Buratta et al. 2020, Westman et al. 2022).

From the fungal perspective, epithelial cell invasion of *C. albicans* can be viewed as an immune evasion strategy. Mice infected with an *als3*Δ/Δ*hyr1*Δ/Δ double mutant exhibit reduced fungal burden due to decreased epithelial cell invasion and increased susceptibility to neutrophil killing. However, when neutrophils and monocytes are depleted, the fungal burden in mice infected with the double mutant is comparable to that in mice infected with the wild-type strain (Phan et al. 2023). In accordance, inhibition of the endocytosis receptors EGFR/HER2, c-Met or AhR in a murine model leads to a decreased oral fungal burden, ameliorating oropharyngeal candidiasis (Zhu et al. 2012, Solis et al. 2017, Phan et al. 2023). This suggests that *C. albicans* can exploit epithelial invasion to evade recognition by immune cells, however, when epithelial cell invasion is reduced, fungal cells are better recognized by immune cells and can clear *C. albicans*, thereby improving disease outcome (Zhu et al. 2012, Solis et al. 2017, Phan et al. 2023). In contrast, mice with knockdown or inhibition of EphA2 and infected with *C. albicans* experience increased fungal burden with larger fungal lesions and deeper tissue invasion (Swidergall et al. 2017), suggesting a beneficial role for EphA2-induced endocytosis in controlling fungal spread. The outcome of epithelial uptake also depends on the infection site (Liu et al. 2011). For example, mice infected with a *C. albicans* mutant lacking a key element of intracellular vesicle trafficking (*vps51*Δ/Δ) survive and show decreased fungal burden in the kidney and liver compared to mice infected with the wild-type strain. However, fungal burden in the brain is significantly increased (Liu et al. 2011). Subsequent experiments revealed that adherence and invasion of *vps51*Δ/Δ to brain epithelial cells is increased due to an elevated surface expression of Als3, which directly interacts with gp96 on brain epithelial cells, leading to an increased fungal uptake. Therefore, increased brain invasion appears beneficial for murine survival as mice also had no signs of neurological disease and clear *C. albicans* from the brain within four days (Liu et al. 2011).

## Aspergillus fumigatus

### Adherence of *A. fumigatus* to airway epithelial cells

**Figure 2:**
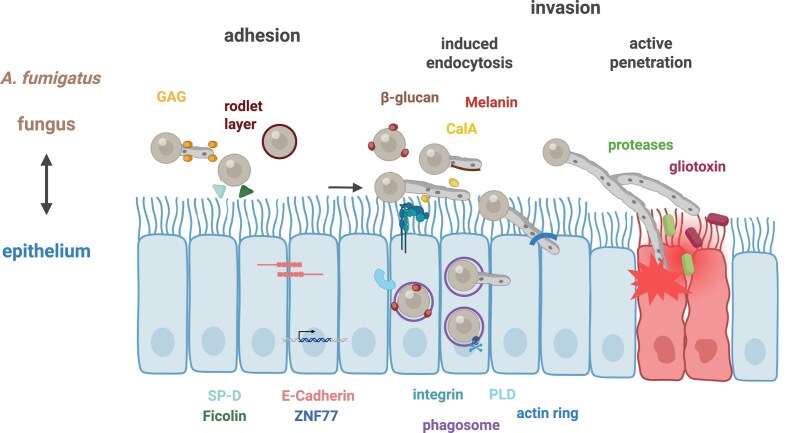
Epithelial and *A. fumigatus* mediators involved in adhesion and invasion. Fungal and host factors involved in adhesion, invasion, and host activities, as discussed in the text, are indicated. The symbols are shown in the same colours as their corresponding names and abbreviations.

One of the first steps in infections caused by *A. fumigatus* is the adhesion of conidia to host cells. Unlike the endogenous infections of *C. albicans* which occurs at multiple mucosal surfaces, infections with *A. fumigatus* are exogenous and primarily occur *via* inhalation of spores into the lung. One of the frontline defence mechanisms of the airway epithelium is trapping inhaled pathogens, such as *A. fumigatus*, in mucus which is then cleared *via* ciliary beating. Alteration in motile cilia structure is associated with lung diseases such as COPD, asthma or cystic fibrosis (Whitsett 2018, Petit et al. 2023). Following inhalation, conidia adhere to airway epithelial cells, initiating swelling, germination, and hyphal growth as the early stages of infection (Takahashi-Nakaguchi et al. 2018). Multiple fungal and host factors influence *A. fumigatus* adherence to the respiratory epithelium (Fig. 2). Similar to *C. albicans*, the fungal cell wall plays a critical role in adherence and recognition by epithelial cells, and its composition varies depending on the fungal morphology (Latgé et al. 2017). The outer cell wall of *A. fumigatus* conidia is composed of a layer of dihydroxynaphtalene (DHN)-melanin covered by a hydrophobic rodlet layer, whereas the outer layer of hyphae can consist of α- and/or β-glucan, chitin, galactomannan and galactosaminogalactan (GAG), with the composition influenced by glucose and oxygen availability (Gow et al. 2017, Latgé et al. 2017, Hopke et al. 2018). These components are important for recognition by epithelial cells. DHN-melanin is recognized by MelLac (Stappers et al. 2018), whereas β-glucan is recognized by Dectin-1 in close interaction with TLR2 (Brown and Gordon 2001, Brown et al. 2003, Underhill 2007, Sun et al. 2012). The conidia surface-exposed heat shock protein HscA binds to the alveolar epithelial cell protein p11 leading to subsequent prevention of phagosome maturation, an important immune evasion strategy (Jia et al. 2023). For example, the cell wall of hypo-adherent Δ*medA* and Δ*stuA A. fumigatus* mutant cells has been shown to contain reduced levels of N-acetylgalactosamine (GalNAc), a key component of GAG (Sheppard et al. 2005, Gravelat et al. 2010, Gravelat et al. 2013). The adherence deficit of Δ*medA*, Δ*stuA* and Δ*uge3*, a strain with no detectable GAG or GalNAc levels, to plastic surfaces is rescued by supplementing GAG but not zymosan, a β-glucan-rich fungal cell wall preparation. Additionally, immunofluorescent microscopy showed a dose-dependent binding of GAG to alveolar epithelial cells (Gravelat et al. 2013). These findings underscore the importance of the *A. fumigatus* cell wall, especially GAG, in adherence to epithelial cells.

As noted earlier, E-cadherin is highly expressed on certain epithelial cell surfaces (or between epithelial cells), including alveolar epithelial cells, where it facilitates calcium-dependent binding by *A. fumigatus* (Xu et al. 2012). Three *A. fumigatus*-expressed proteins have been shown to bind E-cadherin: the protease zymogen AFUA_8G07080 and the outer membrane proteins AfA24A6.130c and AFUA_6G02870 (Xu et al. 2016). *A. fumigatus* adherence to alveolar epithelial cells is significantly reduced when E-cadherin is downregulated. However, adhesion is not completely blocked, indicating that while E-cadherin plays a role in *A. fumigatus* adhesion to airway epithelial cells (at least *in vitro*), additional fungal adhesive molecules are likely involved in early *A. fumigatus*-airway epithelial cell interactions (Xu et al. 2016).

In response to fungal stimulation, alveolar epithelial cells secrete surfactant protein D (SP-D) and A- and H-ficolin, which bind and opsonize *A. fumigatus* conidia, enhancing fungal recognition and killing. The binding of H-ficolin to *A. fumigatus* is calcium-dependent, whereas A-ficolin and SP-D bind in a calcium-independent manner (Bidula et al. 2013, Bidula et al. 2015, Ordonez et al. 2019, Wong et al. 2022).

Multiple polymorphisms in genes involved in the innate or adaptive immunity against *A. fumigatus* have been associated with genetically predisposition to ABPA and worsen disease outcome (Saxena et al. 2003, Knutsen et al. 2006, Vaid et al. 2007, Carvalho et al. 2008, Overton et al. 2016, Gago et al. 2018, Kanaujia et al. 2022, Xu et al. 2023, Kanaujia et al. 2024). For example, a genetic variation in bronchial epithelial cells introducing a premature stop codon in the transcription factor ZNF77 causes increased protein secretion, facilitating pathogen binding and invasion such as collagen, ficolins, cofilin and lectin (Gago et al. 2018). This mutation leads to increased adhesion, invasion, germination, and growth of *A. fumigatus* during interaction with epithelial cells (Gago et al. 2018).

### *A. fumigatus* invasion into epithelial cells

Similar to *C. albicans*, professional phagocytes are the primary cell types responsible for clearing *A. fumigatus* from the lung (Shlezinger et al. 2017, Zhu et al. 2020). Nonetheless, airway epithelial cells can also internalize and kill *A. fumigatus* conidia, albeit to a lesser extent than macrophages. A murine macrophage cell line internalizes 90% of conidia at 3 hpi, whereas alveolar epithelial cells internalize only 30% of conidia (Wasylnka and Moore 2002). Further, <1% of the initial conidial inoculum survived inside macrophages at 12 hpi, whereas 3% of internalized conidia remained alive inside alveolar epithelial cells after 24 h (Wasylnka and Moore 2003). In a leukopenic mouse model, 3.5% of alveolar epithelial cells internalized *A. fumigatus* conidia at 8 hpi, and nearly all internalized conidia (97%) were killed at 16 hpi, highlighting the importance of epithelial cells in *A. fumigatus* clearance *in vivo* (Bertuzzi et al. 2024).

In addition to alveolar epithelial cells, bronchial epithelial cells internalize 20%–40% of conidia at 6 hpi, depending on the bronchial epithelial cell line (Gomez et al. 2010, Clark et al. 2019). Resting and swollen conidia, as well as germlings, are taken up by induced endocytosis (Wasylnka and Moore 2002, Han et al. 2011, Bertuzzi et al. 2014, Jia et al. 2014, Bao et al. 2015, Zhang et al. 2019). However, *A. fumigatus* hyphae can also actively penetrate epithelial tissue, secreting proteases and secondary metabolites that cause epithelial damage (Wasylnka and Moore 2003, Kogan et al. 2004, Bertuzzi et al. 2014, Fernandes et al. 2018, Seidel et al. 2020, Rahman et al. 2022, Okaa et al. 2023).

### Epithelial cells endocytose *A. fumigatus*

Following adhesion to epithelial cells, *A. fumigatus* can initiate its own internalization by host cells (Fig. 2). Invasion by nonprofessional phagocytic cells may serve as an immune evasion strategy by *A. fumigatus*. The host cell cytoskeleton is required for the internalization of *A. fumigatus* conidia into airway epithelial cells. Dynamic activities of the cytoskeleton drive the invagination of the host cell membrane and the uptake of conidia (DeHart et al. 1997, Paris et al. 1997, Wasylnka and Moore 2002, Wasylnka and Moore 2003, Zhang et al. 2005, Bertuzzi et al. 2019). The central regulators of cellular actin dynamics, namely GTPases (Rho, Rac, and Cdc42) and cofilin, are controlled by the cellular signal modulator phospholipase D (PLD) (Han et al. 2011). Human PLD is activated by *A. fumigatus* hyphae and, to an even greater extent, by swollen conidia, leading to increased internalization at these morphological stages. In contrast, resting conidia neither activate PLD nor undergo internalization (Han et al. 2011).

Increased PLD activity is also observed following stimulation with the fungal cell wall component β-1,3-glucan, an important PAMP, and was dependent on the correlating PRR dectin-1. Dectin-1 plays a key role in antifungal immunity by facilitating fungal recognition and killing (Brown and Gordon 2001, Brown et al. 2003, Han et al. 2011, Bertuzzi et al. 2014). In line with this, β-1,3-glucan is masked during most fungal growth stages (Gow et al. 2017, Hopke et al. 2018). For example, knockout mutants lacking the key fungal cell wall regulator pacC showed altered β-glucan distribution, leading to reduced PLD stimulation and fungal internalization during alveolar epithelial cell infection compared to the wild-type strain (Bertuzzi et al. 2014). In accordance, blocking dectin-1 with the monoclonal GE2 antibody reduces *A. fumigatus* internalization by alveolar epithelial cells by ~50% (Han et al. 2011). These findings highlight the importance of β-1,3-glucan in dectin-1-mediated PLD activation and subsequent induction of *A. fumigatus* uptake by airway epithelial cells.

Additional factors modulate PLD activity and the cytoskeletal dynamics to promote *A. fumigatus* internalization. Gliotoxin, the most abundant mycotoxin secreted by *A. fumigatus* hyphae, activates cofilin, a central regulator of cellular actin dynamics. Inhibiting LIM domain kinase 1 (LIMK1, upstream of cofilin) or Rho kinase (ROCK, upstream of LIMK1) reduces phosphorylation and redistribution of cofilin following gliotoxin treatment, thereby inhibiting the uptake of swollen *A. fumigatus* conidia (Bao et al. 2015, Zhang et al. 2019). Interestingly, invasion of Δ*gliP* mutant cells—lacking a key gene in the gliotoxin biosynthetic cluster—into airway epithelial cells was decreased by ~40% and associated with gliotoxin-induced PLD activity. In contrast, the phagocytosis of Δ*gliP* by macrophages was increased by ~60%, also correlating with the absence of gliotoxin-induced PLD activity (Jia et al. 2014). Similarly, mutants lacking the *pskP* enzyme, which is required for DHN-melanin synthesis, exhibit increased uptake by macrophages but reduced uptake by airway epithelial cells (Thywissen et al. 2011, Amin et al. 2014). These findings suggest that gliotoxin and DHN-melanin may contribute to immune evasion by promoting *A. fumigatus* invasion of airway epithelial cells while inhibiting uptake by macrophages (Amin et al. 2014, Jia et al. 2014).

Similarly, inhibition of the small Rho family GTPases Cdc42 or RhoA blocks actin cytoskeleton re-arrangement induced by gliotoxin, whereas inhibition of Rac1 does not (Zhang et al. 2019). As mentioned above, β-1,3-glucan is important for the uptake of *A. fumigatus* germlings by alveolar epithelial cells by activating PLD (Han et al. 2011). Nonetheless, β-1,3-glucan does not appear to be involved in *A. fumigatus* internalization into alveolar epithelial cells mediated by cofilin (Bao et al. 2015). Additionally, gliotoxin treatment does not alter the localization or expression of the tight junction protein ZO-1 and E-cadherin (Zhang et al. 2018). This suggests that gliotoxin specifically targets central regulators of cellular actin dynamics. Nonetheless, the hydrophobic rodlet layer of *A. fumigatus* resting conidia prevents cofilin-induced internalization by alveolar epithelial cells (Bao et al. 2015).

*In silico* proteome analyses have identified the thaumatin protein CalA as a potential adhesion protein of *A. fumigatus*. Recombinant AfCalAp exhibits significant binding to laminin, murine cells, and swollen conidia (Upadhyay et al. 2009). However, a subsequent study reveals that CalA was not required for adhesion to but instead facilitates endocytosis of *A. fumigatus* by alveolar epithelial cells. This process occurs independently of GAG levels and β-1,3-glucan masking. Internalization *via* CalA is initiated through its binding to the host integrin α_5_β_1_ (Liu et al. 2016).

### *A. fumigatus* actively penetrates epithelial cells in a nonlytic and lytic manner

Following internalization by airway epithelial cells, *A. fumigatus* conidia are trafficked into acidic phagolysosomes, a process that may involve the *trans*-Golgi network and/or vesicle transport to the plasma membrane (Clark et al. 2019). Importantly, this process significantly reduces conidia viability, as only 3% of internalized conidia survive inside alveolar epithelial cells after 16 or 24 h (Wasylnka and Moore 2003, Bertuzzi et al. 2024). In contrast to spore-containing phagosomes, 60% of phagosomes containing germlings are not acidified, thereby enabling hyphae formation (Wasylnka and Moore 2003, Seidel et al. 2020). According to (Amin et al. 2014), DHN-melanin and HscA in conidia reduces phagosome acidification, and, therefore, increases intracellular survival (Amin et al. 2014, Jia et al. 2023). This ability of certain *A. fumigatus* conidia to survive within epithelial cells and germinate is closely linked to the inhibition of phagosome maturation. Failure of phagosome maturation can consequently enable the formation of *A. fumigatus* hyphae, which escape from epithelial cells into the extracellular space without causing epithelial cell damage (Wasylnka and Moore 2003, Seidel et al. 2020). Similar to the tunnelling events described for *C. albicans* hyphae, *A. fumigatus* hyphae can extend into adjacent epithelial cells while remaining surrounded by the plasma membrane of the recipient cell (Seidel et al. 2020).

Independent of uptake by airway epithelial cells, extracellular conidia can contribute to pathogenic events by causing epithelial cell detachment (Kogan et al. 2004, Bertuzzi et al. 2014, Rahman et al. 2022, Okaa et al. 2023). Failure to clear extracellular conidia by immune or epithelial cells can result in hyphae formation and active penetration of epithelial cells. Approximately 15% of bronchial epithelial cells were penetrated by *A. fumigatus* hyphae using osmotic pressure at 16 hpi (Fernandes et al. 2018). This was accompanied by the formation of actin rings surrounding penetrating hyphae and the release of pro-inflammatory cytokines (Fernandes et al. 2018). However, penetration is not necessarily associated with epithelial cell damage, indicating that bronchial epithelial cells have evolved protective mechanisms against invading hyphae (Fernandes et al. 2018).

Epithelial cell damage becomes detectable at 12 hpi *in vitro* and increases with rising multiplicity of infection. Secreted compounds from *A. fumigatus* contribute to epithelial damage, since culture filtrates harvested from *A. fumigatus* broth cultures after 48 h cause rapid time- and dose-dependent epithelial cell damage at 2 h post-challenge. The above-mentioned transcription factor PacC modulates the expression of over 250 secreted proteins, including gliotoxin (Bertuzzi et al. 2014). Damage of epithelial cells is significantly reduced during infections with *ΔpacC, ΔgliP* or *ΔprtT* (major regulator of protease secretion) mutants (Okaa et al. 2023). In conclusion, epithelial cell damage and death are initiated by conidia-induced epithelial cell detachment during early infection stages, whereas soluble fungal products and host immune responses drive lytic cell death at later infection stages (Okaa et al. 2023).

### Is epithelial uptake of *A. fumigatus* beneficial for the host?

Nonprofessional phagocytic airway epithelial cells predominantly act as barriers and play a crucial role in communicating with and alerting professional phagocytes in response to fungal invasion and damage. Unlike *C. albicans*, invasion of *A. fumigatus* fungal elements *via* induced endocytosis may be beneficial for the host, as airway epithelial cells have been shown to kill internalized conidia both *in vitro* and *in vivo* (Wasylnka and Moore 2003, Amin et al. 2014, Bertuzzi et al. 2024). Nonetheless, it remains to be elucidated whether *A. fumigatus* uptake by airway epithelial cells represents a pathogenic event or a protective mechanism for the host. In cystic fibrosis patients, internalization of *A. fumigatus* conidia is associated with increased host survival (Chaudhary et al. 2012). Conversely, in invasive aspergillosis murine models, internalization leads to increased fungal burden and decreased host survival (Gravelat et al. 2013, Liu et al. 2016, Zhang et al. 2019). In a cystic fibrosis model, a mutation in the cystic fibrosis transmembrane conductance regulator reduces conidial internalization by epithelial cells, resulting in increased murine fungal burden and exacerbation of cystic fibrosis symptoms (Chaudhary et al. 2012). In contrast, mice infected with *A. fumigatus* knockout mutants lacking the ability to produce GAG (Δ*uge3*), gliotoxin (Δ*gliP*), or bind the host integrin α_5_β_1_ (Δ*calA*) exhibit longer survival and decreased fungal burden compared to wild-type infected mice (Gravelat et al. 2013, Liu et al. 2016, Zhang et al. 2019).

## Conclusion

To conclude, adhesion to epithelial cells is a prerequisite of epithelial invasion by fungal elements of *C. albicans* or *A. fumigatus*. While yeast cells of *C. albicans* are noninvasive, conidia of *A. fumigatus* can be internalized by epithelial cells. Induced endocytosis of *C. albicans* requires germ tube formation. While hyphae of both *C. albicans* and *A. fumigatus* can actively penetrate epithelial cells, these invasion processes, *per se*, are not necessarily associated with host cell damage. Induced endocytosis of *A. fumigatus* by airway epithelial cells even result in fungal killing. To cause maximum host cell damage, both *C. albicans* and *A. fumigatus* hyphae rely on the expression of hyphal associated factors, such as the *C. albicans* peptide toxin candidalysin or *A. fumigatus* secreted proteases and gliotoxin. Therefore, while *C. albicans* and *A. fumigatus* share fundamental mechanisms of adhesion and invasion as initial processes of mycoses, they also exhibit remarkable differences in their strategies and outcomes.
